# Genotypic resistance determined by whole genome sequencing versus phenotypic resistance in 234 *Escherichia coli* isolates

**DOI:** 10.1038/s41598-023-27723-z

**Published:** 2023-01-09

**Authors:** R. Vanstokstraeten, D. Piérard, F. Crombé, D. De Geyter, I. Wybo, A. Muyldermans, L. Seyler, B. Caljon, T. Janssen, T. Demuyser

**Affiliations:** 1 Department of Microbiology and Infection Control, Vrije Universiteit Brussel (VUB), Universitair Ziekenhuis Brussel (UZ Brussel), Brussels, Belgium; 2 Department of Infectious Diseases, Universitair Ziekenhuis Brussel (UZ Brussel), Brussels, Belgium; 3 Brussels Interuniversity Genomics High Throughput Core (BRIGHTcore) Platform, Vrije Universiteit Brussel (VUB), Universitair Ziekenhuis Brussel (UZ Brussel), Brussels, Belgium

**Keywords:** Antibiotics, Antimicrobial resistance, Clinical microbiology

## Abstract

Whole genome sequencing (WGS) enables detailed characterization of bacteria at single nucleotide resolution. It provides data about acquired resistance genes and mutations leading to resistance. Although WGS is becoming an essential tool to predict resistance patterns accurately, comparing genotype to phenotype with WGS is still in its infancy. Additional data and validation are needed. In this retrospective study, we analysed 234 *E. coli* isolates from positive blood cultures using WGS as well as microdilution for 11 clinically relevant antibiotics, to compare the two techniques. We performed whole genome sequencing analyses on 234 blood culture isolates (genotype) to detect acquired antibiotic resistance. Minimal inhibitory concentrations (MIC) for *E. coli* were performed for amoxicillin, cefepime, cefotaxime, ceftazidime, meropenem, amoxicillin/clavulanic acid, piperacillin/tazobactam, amikacin, gentamicin, tobramycin, and ciprofloxacin, using the ISO 20776-1 standard broth microdilution method as recommended by EUCAST (phenotype). We then compared the two methods for statistical ‘agreement’. A perfect (100%) categorical agreement between genotype and phenotype was observed for gentamicin and meropenem. However, no resistance to meropenem was observed. A high categorical agreement (> 95%) was observed for amoxicillin, cefepime, cefotaxime, ceftazidime, amikacin, and tobramycin. A categorical agreement lower than 95% was observed for amoxicillin/clavulanic acid, piperacillin/tazobactam, and ciprofloxacin. Most discrepancies occurred in isolates with MICs within ± 1 doubling dilution of the breakpoint and 22.73% of the major errors were samples that tested phenotypically susceptible at higher antibiotic exposure and were therefore considered as ‘not resistant’. This study shows that WGS can be used as a valuable tool to predict phenotypic resistance against most of the clinically relevant antibiotics used for the treatment of *E. coli* bloodstream infections.

## Introduction

*Escherichia coli* (*E. coli*) is a Gram-negative, rod-shaped, facultative anaerobic bacterium^1^. It is the most abundant facultative anaerobic bacterium in the human gastrointestinal tract^2^. Most of the non-pathogenic *E. coli* strains have, like many other intestinal bacteria, a commensal relationship with humans. By producing certain chemical substances and competing for nutrients, this species protects us from potential pathogens. However, this commensal relationship can also go wrong when this bacterium reaches the bloodstream via the urinary tract or gut translocation and causes a bloodstream infection^3^. *E. coli* is the most common species of the order of *Enterobacterales* to cause bloodstream infections*.* Strains capable of causing bloodstream infections are known as extraintestinal pathogenic *E. coli* (ExPEC), defined by some virulence factors like *papC, iutA, papA_F7-2,* and *kpsMII,* specialized to invade and survive in the bloodstream. These ExPEC strains differ from diarrheagenic *E. coli* in their epidemiologic, phylogenetic, and virulence factor profiles^4^.

Antibiotic resistance is a bacterial characteristic that can be intrinsic or acquired*.* Of interest in this study is the acquisition of resistance to antibiotics by intrinsically susceptible *E. coli* species. Bacteria can become resistant through mutations within acquired resistance genes and mutations in cellular genes. These mutations can lead to reduced target affinity, alterations of regulatory networks that control the expression of resistance-regulatory proteins, and reduced access to the bacterial membrane. *E. coli* has a great capacity to accumulate resistance genes through horizontal gene transfer. Plasmids and other mobile genetic elements such as gene cassettes in class one and class two integrons and transposons play a major role in the horizontal gene transfer of resistance genes^5,6^.

Genomic analysis, and more specifically whole genome sequencing (WGS), enables detailed characterization of the bacterium at single nucleotide resolution. It provides data on the serotype, pathotype, sequence type (ST), acquired antibiotic resistance, and virulence factors. WGS is therefore a very powerful tool for routine surveillance and outbreak investigation^7^. Although WGS could become an essential tool to predict resistance patterns, clinical laboratories still rely on dilution and diffusion susceptibility testing to guide clinical therapy. Bringing a sequencing-based approach into the routine would be very costly, and requires robust bioinformatics tools and experienced personnel. In addition, genotyping antibiotic resistance is still in its infancy so additional data and validation are eagerly awaited^8^.

In our previous research paper, we performed WGS and broth dilution for amoxicillin/clavulanic acid on *E. coli* blood isolates^9^. We described the acquired beta-lactamase genes and highlighted the low level of agreement between EUCAST and CLSI methodologies when performing minimal inhibitory concentration (MIC) testing of amoxicillin/clavulanic acid.

In the current research paper, we performed broth dilution for 10 additional antibiotics and analysed the acquired resistance genes and the known chromosomal point mutations leading to resistance on the same isolates using BioNumerics v.8.1 (Applied Maths, Biomérieux, Belgium), which is a software used for functional genotyping based on the most recent Resfinder and Pointfinder databases (http://www.genomicepidemiology.org/services/) combined with private knowledge. In this study, we described the acquired resistance mechanisms present in 234 *E. coli* isolates. We then looked at whether genotypic resistance matched with phenotypic resistance obtained through broth dilution, for 11 clinically relevant antibiotics to treat *E. coli* bloodstream infections.

## Material and methods

The UZ Brussel is a tertiary care center with over 700 beds. Two hundred and thirty-four (234) non-duplicate clinical isolates were analysed. We chose *E. coli* strains isolated from clinical blood samples, randomly selected between 1985 and 2018. All isolates were stored at − 80 °C. The isolates were distributed over the years as follows: 1985 (n = 10), 1990 (n = 11), 1995 (n = 12), 2000 (n = 34), 2005 (n = 33), 2010 (n = 33), 2015 (n = 51), and 2018 (n = 50).

### MIC testing

MIC was performed for amoxicillin (1–32 µl/ml), cefepime (0.5–32 µg/ml), cefotaxime (0.5–8 µg/ml), ceftazidime (0.5–32 µg/ml), meropenem (0.12–32 µl/ml), amoxicillin/clavulanic acid (1/0.5–32/16 µl/ml), piperacillin/tazobactam (1/4–64/4 µg/ml), amikacin (4–32 µg/ml), gentamicin (0.5–8 µl/ml), tobramycin (1–16 µg/ml) and ciprofloxacin (0.06–8 µl/ml) by using the ISO 20776-1 standard broth microdilution method as recommended by EUCAST^10^. We used Sensititre™ Custom Plates according to EUCAST guidelines also. We used the EUCAST clinical breakpoints to interpret phenotypic susceptibility results^11^. For statistical analysis, the EUCAST’s phenotypic category ‘susceptible, increased exposure’ was combined with the ‘susceptible’ category, as recommended by this organization.

### Phenotypic ESBL-detection

The EUCAST disk diffusion method for phenotypic detection of extended-spectrum beta-lactamases (ESBL) was performed on Mueller-Hilton agar (I2A, Montpellier, France) with ceftazidime, ceftriaxone, cefepime, and clavulanic acid. The Mueller-Hilton agar plates were incubated for 24 h at 37 °C. After incubation, the zone of inhibition was measured by SIRscan^®^ (I2A, Montpellier, France). The EUCAST algorithm was used to interpret the disk diffusion diameters^11^.

### DNA isolation and whole genome sequencing

Two different methods were used to perform WGS of the *E. coli* isolates. Genomic DNA was extracted using the Dneasy blood & tissue kit (Qiagen, Hilden, Germany) for 30 samples, and DNA libraries were prepared via the KAPA Hyper Plus kit (Kapa Biosystems, Wilmington, MA, USA). All libraries were sequenced on a MiSeq instrument (Illumina, San Diego, CA, USA) using the v2 (2 × 250 bp) and v3 (2 × 300 bp) reagent kits. For the remaining 203 samples, genomic DNA was extracted using the Maxwell RSC Cell DNA purification kit (Promega Corporation, Madison, USA). Fragmentation of 500 ng of genomic DNA was carried out using the NEBNext^®^ Ultra™ II FS module. Sequencing libraries, with an insert size of on average 550 bp, were prepared using the KAPA Hyper Plus kit (Kapa Biosystems, Wilmington, USA) and a Pippin Prep (Sage Science, Beverly, MA, USA) size with the CDF1510 1.5% agarose dye-free cassette selection. In order to avoid PCR bias, the PCR amplifications step was omitted and a 500 ng input of genomic DNA was used. After equimolar pooling, libraries were sequenced on a Novaseq 6000 instrument (Illumina, San Diego, CA, USA) using the NovaSeq 6000 SP Reagent Kit (500 cycles) generating 2 × 250 bp reads. To achieve this, the library was denatured and diluted according to the manufacturer’s instructions. A 1% PhiX control library was included in each sequencing run. Sequence quality was assessed with FastQC (version 0.11.4) software (https://www.bioinformatics.babraham.ac.uk/projects/fastqc/).

De novo assembly was performed using SPAdes genome assembler in BioNumerics.

The quality of the sequence read sets and the de novo assemblies were verified using the quality assessment tool available in BioNumerics.

The sequenced data were analysed for acquired and mutational resistance and serotypes using the *E.coli* genotyping tool available in BioNumerics v.8.1 (Applied Maths, Biomérieux, Belgium). The presence of resistance genes was determined with a minimum percentage sequence identity (ID) threshold of 95% and a minimum length for sequence coverage of 95%. This genotyping tool is based on publicly available databases on the Center of Genomic Epidemiology (http://www.genomicepidemiology.org/). Assembled genomes were analyzed using the MLST Achtman scheme (7 MLST loci) available in BioNumerics v.8.1.

### Statistical analysis

Broth dilution is considered the Gold standard for determining susceptibility in all statistical analyses (categorical agreement, errors, sensitivity, specificity, and predictive values). Major errors are defined as resistant genotype and susceptible phenotype. Very major errors are defined as susceptible genotype and resistant phenotype.

### Ethics approval

The Medical Ethics Committee of UZ Brussel/VUB reviewed and approved the documents concerning our project from the ethical, legal, and medical points of view (Reference: 1432021000569). We confirm that all methods were carried out in accordance with relevant guidelines and regulations. The Ethics Committee of UZ Brussels/VUB decided no informed consent was needed from the subjects.

## Results

### Resistance

**Figure 1:**
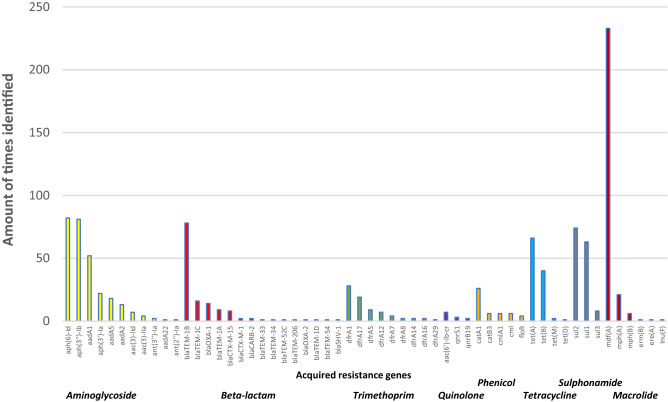
Identified acquired resistance genes from 234 *E. coli* isolates isolated from bloodstream infections between 1985 and 2018. From left to right: resistance genes against aminoglycosides (yellow), beta-lactams (pink), trimethoprim (green), quinolones (purple), phenicol (orange), tetracyclines (blue), sulphonamides (grey) and macrolides (red). Genes responsible for resistance to the 11 tested antibiotics are summarized in the Supplementary Data 3.

**Figure 2:**
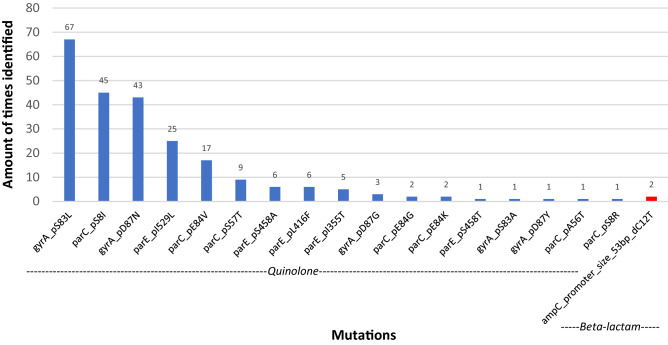
Identified mutational resistance based on mutations with a known phenotype from 234 *E. coli* isolates from bloodstream infections between 1985 and 2018. Mutations in the gyrA and parC genes can result in (fluoro) quinolone resistance (blue), mutations in ampC-promotor genes can results in beta-lactam resistance (red). Mutations responsible for resistance to the 11 tested antibiotics are summarized in the Supplementary Data 3.

In total, 57 different acquired resistance genes and 18 mutations associated with known resistance phenotypes for aminoglycosides, beta-lactams, trimethoprim, quinolones, phenicol, tetracyclines, sulphonamides, and macrolides are summarized in Figs. 1 and 2. The genes and mutations responsible for resistance genotypes to the 11 tested antibiotics are listed in the supplementary data.

One-hundred-thirty-one of the 234 (55.98%) *E. coli* isolates showed genotypic resistance against amoxicillin, 23/234 (9.83%) against cefepime, 15/234 (6.41%) against cefotaxime, 15/234 (6.41%) against ceftazidime, 0/234 (0%) against meropenem, 21/234 (8.97%) against amoxicillin/clavulanic acid, 19/234 (8.12%) against piperacillin/tazobactam, 6/234 (2.56%) against amikacin, 12/234 (5.13%) against gentamicin, 16/234 (6.88%) against tobramycin and 70/234 (29.91%) against ciprofloxacin.

One-hundred-thirty-six of the 234 (58.12%) *E. coli* isolates tested phenotypically resistant for amoxicillin, 10/234 (4.27%) for cefepime, 12/234 (5.13%) for cefotaxime, 9/234 (3.85%) for ceftazidime, 0/234 (0%) for meropenem, 92/234 (39.32%) for amoxicillin/clavulanic acid, 17/ 234 (7.26%) for piperacillin/tazobactam, 0/234 (0%) for amikacin, 12/234 (5.13%) for gentamicin and 13/234 (5.56%) for tobramycin and 45/233 (19.23%) for ciprofloxacin.

Our set observed no phenotypic resistance against meropenem, so meropenem was excluded from further analysis. The genotypic and phenotypic resistance characteristics of all 234 strains are summarized in Supplementary Data 1 and 2.

### Comparing genotype with phenotype

We found an average categorical agreement of 94.13% between our genotypic tests and the reference phenotypic tests. A perfect (100%) categorical agreement between genotypes and phenotypes was observed for gentamicin and meropenem. A high categorical agreement (> 95%) was observed for amoxicillin, cefepime, cefotaxime, ceftazidime, amikacin, and tobramycin. A categorical agreement lower than 95% was observed for amoxicillin/clavulanic acid, piperacillin/tazobactam, and ciprofloxacin.

Major errors were observed for all antibiotics, with the exclusion of amoxicillin/clavulanic acid and gentamicin. Although we have to mention that 15/66 (22.73%) of the major errors tested phenotypically ’susceptible, increased exposure’ and were therefore considered as not resistant. Very major errors were only observed in amoxicillin, amoxicillin/clavulanic acid and piperacillin/tazobactam.

A perfect (100%) sensitivity was found for all antibiotics, with the exclusion of amoxicillin, amoxicillin/clavulanic acid, piperacillin/tazobactam, and tobramycin. No sensitivity could be calculated for amikacin due to the lack of phenotypic resistant isolates in our set. High specificity (> 95%) was found for all antibiotics, with the exclusion of ciprofloxacin. Noticeably, 14.89% of the strains phenotypically susceptible to ciprofloxacin harbored one mutation or acquired gene associated with resistance to ciprofloxacin. All 45 resistant strains harbored more than one mutation or acquired resistance gene associated with resistance to ciprofloxacin.

The positive predictive value was found to be very diverse in our dataset: 99.24% for amoxicillin, 36.36% for cefepime, 80.00% for cefotaxime, 60.00% for ceftazidime, 100% for amoxicillin/clavulanic acid, 50.00% for piperacillin/tazobactam, 100% for gentamicin, 75.00% for tobramycin and 64.29% for ciprofloxacin. No positive predictive values could be calculated for amikacin due to the lack of phenotypic-resistant isolates in our dataset. A perfect (100%) negative predictive value was found for all antibiotics with the exclusion of amoxicillin/clavulanic acid, piperacillin/tazobactam, and ciprofloxacin.

**Figure 3:**
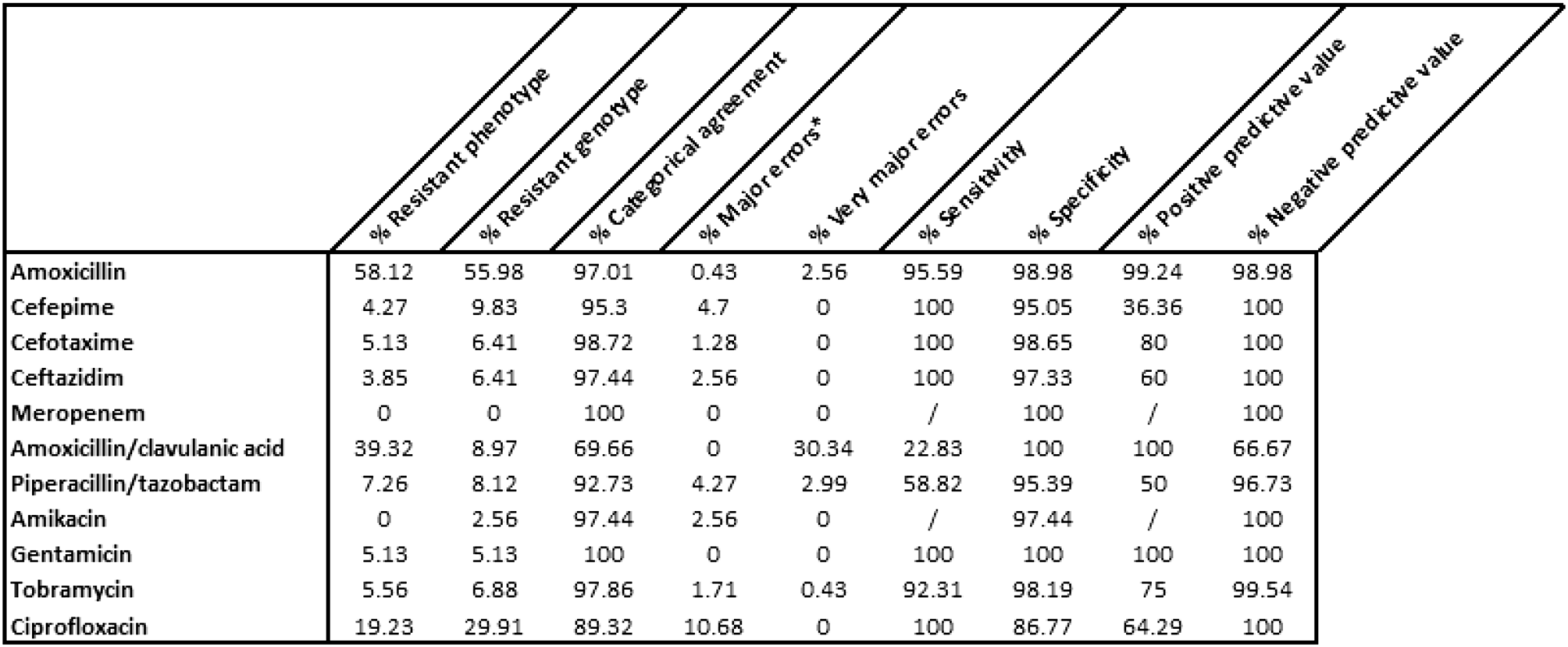
Summary of the results of the 234 analyzed *E. coli* isolates. Meropenem was excluded due to the lack of resistant isolates. *Of note, 22.73% of the major errors tested phenotypically as ‘susceptible at higher exposure’, and were therefore considered as not resistant.

All the above-mentioned results are summarized and visualized in detail in Fig. 3 and Supplementary Data 4.

### Extended-spectrum beta-lactamases

Twelve of the 234 (5.13%) isolates were considered ESBL-positive according to the disk diffusion test. In 11/12 (91.67%) of these isolates, an ESBL-gene was identified, with the *blaCTX-M-15* gene being the most prevalent one, found in eight of the 12 (66.67%) isolates. Two of the 12 (16.67%) isolates carried a *blaCTX-M-1* gene and one of the 12 (8.33%) isolates carried a *blaTEM-52C* gene. In the one isolate that carried no ESBL-gene but tested phenotypically positive for ESBL, we found a point mutation in the AmpC-promotor known to induce resistance to ceftazidime, cefepime, and clavulanic acid and therefore possibly explaining the ESBL-phenotype. However, this mutation does not cause resistance to ceftriaxone. All the isolates carrying an ESBL-gene tested phenotypically positive for ESBL. If disk diffusion is considered the Gold standard for ESBL-testing, a categorical agreement was found in 11/12 (91.67%) of our samples. We observe an association (P < 0.05 when performing a Fisher Exact test) between ESBL genes and the presence of blaOXA-1, a gene responsible for resistance to the above-mentioned antibiotics. Fourteen of the 234 (5.13%) isolates carried a blaOXA-1 gene, of which four were found in ESBL-positive strains and eight were found in the ESBL-negative strains. Fourteen of the 234 (5.13%) isolates carried a blaOXA-1 gene, four (33.33%) of which were found in the 12 ESBL-positive strains.

### Multi-locus sequence type and serotype

Eighty-two different STs, 82 different O- and 28 different H-serotypes were identified among the 234 isolates. The five dominant STs were: 26 (11.11%) ST73, 25 (10.68%) ST131, 21 (8.97%) ST69, 18 (7.69%) ST95, and 15 (6.41%) ST88. The five dominant O-serotypes were: 29 (12.39%) O25, 22 (9.40%) O6, 22 (9.40%) O9, 17 (7.26%) O1, and 14 (5.98%) O15. The five dominant H-serotypes were: 57 (24.36%) H4, 35 (14.96%) H1, 22 (9.40%) H7, 19 (8.12%) H5, and 18 (7.69%) H18. All identified STs and serotypes are listed in the Supplementary Data 1.

## Discussion

In this research paper, we described the utility of WGS for predicting antibiotic resistance against 11 clinically relevant antibiotics to treat *E. coli* bloodstream infections. We identified 57 different acquired resistance genes and 18 resistance-associated mutations with known phenotypes. We found an average categorical agreement of 94.13% between our genotypic tests and the reference phenotypic tests. A perfect (100%) categorical agreement between genotypes and phenotypes was observed for gentamicin and meropenem. A high categorical agreement (> 95%) was observed for amoxicillin, cefepime, cefotaxime, ceftazidime, amikacin, and tobramycin. A categorical agreement lower than 95% was observed for amoxicillin/clavulanic acid, piperacillin/tazobactam, and ciprofloxacin. Most discrepancies occurred in isolates with MICs within ± 1 doubling dilution of the breakpoint. Of note, 22.73% of the major errors/discrepancies tested phenotypically as ‘susceptible at higher exposure’, and were therefore considered as not resistant (Fig. 3).

Bortolaia et al*.* compared the phenotypic resistance of 584 *E. coli* isolates based on MIC against 16 antibiotics, with genotypic resistance based on ResFinder 4.0. They found an overall genotype–phenotype concordance of 97%, ranging from 71.6% for cefepime to 100% for most antibiotics. In our study, the average categorical agreement was lower. However, our study included amoxicillin/clavulanic acid and piperacillin/tazobactam, which they did not^8^.

Tyson et al*.* already reported the utility of WGS for the accurate prediction of antibiotic resistance in *E. coli*. They described more than 30 acquired resistance genes among 76 *E. coli* isolates. The resultant resistance genotypes correlated with 99.6% sensitivity and 97.8% specificity to resistance phenotypes. Overall, our percentages of the categorical agreement are lower than in the work of Tyson et al*.* possibly because we did not retest discrepant results as they did. We also did not make a selection based on multidrug-resistant profiles, which they did: in our study, we included isolates randomly without previous knowledge about their resistance profiles^12^.

Although our results show a high degree of correlation between resistance genotypes and phenotypes for most antibiotics, it is not the case for amoxicillin/clavulanic acid. This is in line with the results of Davies et al. They point out the fact that amoxicillin/clavulanic acid resistance in *E. coli* is rather quantitative than qualitative and that resistance is built up by many different features, resulting in suboptimal concordance when using binary classification, such as phenotypic classifications^13^. The underlying mechanisms resulting in a resistant phenotype are more complex and variable, and other factors than solely the presence of acquired resistance genes or mutations very likely play an important role. For example, an induced AmpC expression^14^. Before obtaining reliable genotypic resistance testing of beta-lactamase inhibitor combinations, such as amoxicillin/clavulanic acid, and to a lesser extent piperacillin/tazobactam, more efforts should be done to reveal the factors determining expression levels of the beta-lactamases.

In the case of ciprofloxacin, as many as 13.23% of strains harboring resistance genes or resistance-associated mutations were phenotypically susceptible. They harbored only one of them, while all strains classified as resistant harbored at least two of them. This was expected, as it is known that resistance to fluoroquinolones requires the accumulation of multiple acquired resistance genes or mutations, including those that alter increased drug efflux^15^. In comparison with dilution and diffusion methods, it is also important to note that genotypic resistance is expressed either as present or absent and does not measure clinical resistance thresholds.

Eleven of the 12 (91.67%) strains with an ESBL-positive phenotype were identified as ESBL-positive with WGS. We observe the co-carriage of blaOXA-1 in ESBL-positive strains, highlighting a link between ESBL-producing bacteria and resistance to amoxicillin/clavulanic acid and piperacillin/tazobactam. Concordant with Livermore et al*.* the ESBL accompanying OXA-1 was always CTX-M-15^16^.

Antibiotic resistance is a growing problem. Rapid and correct selection of an appropriate antibiotic is of great importance in the clinic, especially in severe and invasive infections such as bloodstream infections. Despite the application of WGS in the prediction of phenotypic resistance profiles are fairly well known, the data available on *E. coli* blood isolates is limited. Therefore, additional data like those provided in our study is highly needed. In this study performed on randomly selected blood isolates, we demonstrated that WGS could accurately predict the vast majority of resistance phenotypes against the antibiotics we tested. Most discrepant results were observed for beta-lactamase combinations, mainly amoxicillin/clavulanic acid, suggesting that high levels of beta-lactamase production are involved in low-level resistance. More efforts should be done to better understand the factors determining the expression levels of these enzymes.

A limitation of this study is the low resistance rates to some antibiotics such as meropenem, which was therefore excluded from parts of the analysis. It is important to take into account that this study only focuses on unimicrobial bacterial cultures of *E. coli* as the lead bacterial pathogen in the frame of multimicrobial infections leading to *E. coli* bacteremia. These multispecies communities might critically influence the antibiotic resistance gene expression of the lead pathogen, often resulting in a lack of accuracy of antimicrobial susceptibility testing of one micro-organism to predict the in vivo success or failure of antibiotic therapy^17^.

There is an undeniable potential for WGS-based techniques to replace dilution and diffusion susceptibility testing to guide clinical therapy. Currently, phenotypical susceptibility testing is still much cheaper and faster than performing WGS. However, this will probably change over time since WGS is getting cheaper. WGS also provides a massive amount of additional information that classic dilution and diffusion susceptibility tests do not provide, such as virulence factors and the relatedness of bacterial isolates^18^. This additional information could be of great value to investigate the bacterial origin, for example.

## Conclusion

In conclusion, this study shows that WGS can be used as a valuable tool to predict phenotypic resistance in most of the clinically relevant antibiotics to treat *E. coli* bloodstream infections, with high specificity and sensitivity. However, excessive beta-lactamase expression, exceeding the activity of inhibitors, leads to a lower accuracy of genotypic tests to detect resistance to beta-lactam combinations. Before applying WGS in a clinical context, the genetic basis of those resistance mechanisms should be unraveled.

## Supplementary Information

Supplementary Information.

## Data Availability

The datasets generated and/or analysed during the current study are available in the National Library of Medicine repository. BioProject: PRJNA854358.

## References

[1] Kaper JB, Nataro JP, Mobley HLT (2004). Pathogenic *Escherichia coli*. Nat. Rev. Microbiol..

[2] Mora-Rillo M (2015). Impact of virulence genes on sepsis severity and survival in *Escherichia coli* bacteremia. Virulence.

[3] Leimbach A, Hacker J, Dobrindt U (2013). *E. coli* as an all-rounder: The thin line between commensalism and pathogenicity. Curr. Top. Microbiol. Immunol..

[4] Micenková L (2017). Human Escherichia coli isolates from hemocultures: Septicemia linked to urogenital tract infections is caused by isolates harboring more virulence genes than bacteraemia linked to other conditions. Int. J. Med. Microbiol..

[5] Poirel L, Madec JY, Lupo A, Schink AK, Kieffer N, Nordmann P, Schwarz S (2018). Antimicrobial resistance in *Escherichia coli*. Microbiol. Spectr..

[6] Olorunmola FO, Kolawole DO, Lamikanra A (2013). Antibiotic resistance and virulence properties in *Escherichia coli* strains from cases of urinary tract infections. Afr. J. Infect. Dis..

[7] Rossen JWA, Friedrich AW, Moran-Gilad J, ESCMID Study Group for Genomic and Molecular Diagnostics (ESGMD) (2018). Practical issues in implementing whole-genome-sequencing in routine diagnostic microbiology. Clin. Microbiol. Infect..

[8] Bortolaia V, Kaas RS, Ruppe E, Roberts MC, Schwarz S, Cattoir V, Philippon A, Allesoe RL, Rebelo A, Florensa AF, Fagelhauer L, Chakraborty T, Neumann B, Werner G, Bender JK, Stingl K, Nguyen M, Coppens J, Xavier BB, Malhotra-Kumar S, Westh H, Pinholt M, Anjum MF, Duggett NA, Kempf I, Nykäsenoja S, Olkkola S, Wieczorek K, Amaro A, Clemente L, Mossong J, Losch S, Ragimbeau C, Lund O, Aarestrup FM (2020). ResFinder 4.0 for predictions of phenotypes from genotypes. J. Antimicrob. Chemother..

[9] Vanstokstraeten R, Belasri N, Demuyser T (2021). A comparison of *E. coli* susceptibility for amoxicillin/clavulanic acid according to EUCAST and CLSI guidelines. Eur. J. Clin. Microbiol. Infect. Dis..

[10] International Standard, ISO 20776–1 Susceptibility testing of infectious agents and evaluation of performance of antimicrobial susceptibility test devices. Part 1: Broth micro-dilution reference method for testing the in vitro activity of antimicrobial agents against rapidly growing aerobic bacteria involved in infectious diseases. Accessed 1 May 2022. https://cdn.standards.iteh.ai/samples/70464/9153ed0ea56d45048f666016159924a0/ISO-20776-1-2019.pdf (2019).

[11] EUCAST (2017), EUCAST guidelines for the detection of resistance mechanisms and specific resistance of clinical and/or epidemiological importance pp. 13–20. Accessed 1 May 2022. https://www.eucast.org/resistance_mechanisms/ [Online]. https://www.eucast.org/fileadmin/src/media/PDFs/EUCAST_files/Resistance_mechanisms/EUCAST_detection_of_resistance_mechanisms_170711.pdf (2017).

[12] Tyson GH, McDermott PF, Li C, Chen Y, Tadesse DA, Mukherjee S, Bodeis-Jones S, Kabera C, Gaines SA, Loneragan GH, Edrington TS, Torrence M, Harhay DM, Zhao S (2015). WGS accurately predicts antimicrobial resistance in *Escherichia coli*. J. Antimicrob. Chemother..

[13] Davies TJ, Stoesser N, Sheppard AE, Abuoun M, Fowler P, Swann J, Quan TP, Griffiths D, Vaughan A, Morgan M, Phan HTT, Jeffery KJ, Andersson M, Ellington MJ, Ekelund O, Woodford N, Mathers AJ, Bonomo RA, Crook DW, Peto TEA, Anjum MF, Walker AS (2020). Reconciling the potentially irreconcilable? Genotypic and phenotypic amoxicillin-clavulanate resistance in *Escherichia coli*. Antimicrob. Agents Chemother..

[14] Jacoby GA (2009). AmpC beta-lactamases. Clin. Microbiol. Rev..

[15] Ma G, Wu G, Li X, Wang H, Zhou M (2018). Characterization of ciprofloxacin resistance in laboratory-derived mutants of vibrio parahaemolyticus with qnr gene. Foodborne Pathog. Dis..

[16] Livermore DM, Day M, Cleary P, Hopkins KL, Toleman MA, Wareham DW, Wiuff C, Doumith M, Woodford N (2019). OXA-1 β-lactamase and non-susceptibility to penicillin/β-lactamase inhibitor combinations among ESBL-producing *Escherichia coli*. J. Antimicrob. Chemother..

[17] Tetz G, Tetz V (2022). Overcoming antibiotic resistance with novel paradigms of antibiotic selection. Microorganisms.

[18] De Geyter D, Vanstokstraeten R, Crombé F, Tommassen J, Wybo I, Piérard D (2021). Sink drains as reservoirs of VIM-2 metallo-beta-lactamase-producing Pseudomonas aeruginosa in a Belgian intensive care unit: relation to patients investigated by whole-genome sequencing. J. Hosp. Infect..

